# Yijinjing Qigong intervention shows strong evidence on clinical effectiveness and electroencephalography signal features for early poststroke depression: A randomized, controlled trial

**DOI:** 10.3389/fnagi.2022.956316

**Published:** 2022-08-10

**Authors:** Pingping Sun, Shuaipan Zhang, Linhong Jiang, Zhenzhen Ma, Chongjie Yao, Qingguang Zhu, Min Fang

**Affiliations:** ^1^School of Rehabilitation Science, Shanghai University of Traditional Chinese Medicine, Shanghai, China; ^2^Tuina Department, Yueyang Hospital of Integrated Traditional Chinese and Western Medicine, Shanghai University of Traditional Chinese Medicine, Shanghai, China; ^3^School of Acupuncture-Moxibustion and Tuina, Shanghai University of Traditional Chinese Medicine, Shanghai, China; ^4^Longhua Hospital, Shanghai University of Traditional Chinese Medicine, Shanghai, China; ^5^Department of Tuina Research, Research Institute of Traditional Chinese Medicine in Shanghai, Shanghai, China

**Keywords:** poststroke depression, traditional exercise, Yijinjing Qigong exercise, EEG, brain functional connectivity network

## Abstract

**Objective:**

Although Traditional Chinese Yijinjing Qigong Exercise (YJJQE) as mind–body intervention is popularly used among adults to ameliorate depressive symptoms in China, no randomized controlled trials (RCTs) are available to evaluate the effects of YJJQE in patients with poststroke depression (PSD). This study aims to explore the clinical efficacy and the neurological and psychiatric mechanism in brain network functional connectivity underlying electroencephalography (EEG).

**Materials and methods:**

A total of 60 patients, diagnosed with mild PSD, were randomly (1:1) assigned to YJJQE group (*n* = 30) and control group of routine segmental rehabilitation training group (*n* = 30) for a 60-min exercise session once a day for 3 weeks. All outcome measures were collected at baseline and 3-weeks ending intervention. The primary outcome was the 24-item Hamilton Depression Scale (HAMD-24) score, evaluation at more time points for 1 month of follow-up. The secondary outcomes were EEG data in four frequency domains (δ, θ, α, and β), global efficiency (GE), local efficiency (LE), GE/LE curve [areas under the curve (AUC)], Phase Lag Index (PLI), (HAMD-24) Score and EEG correlation analysis.

**Results:**

All patients showed no significant differences in baseline data. After 3 weeks and 1 month of follow-up, the YJJQE group demonstrated significant decreasing changes compared to the control group on the HAMD-24 scores (*p* < 0.001). Furthermore, the YJJQE group also showed a significant reduction in θ wave, and an increase in both GE and LE. Compared to the control group, the YJJQE Qigong group showed significantly greater functional connectivity in the δ, θ, and β frequency bands in the brain network of the degree of phase synchronization (*p* < 0.001). HAMD-24 Score and EEG correlation analysis negative correlation in the Qigong group θ wave (*p* < 0.001).

**Conclusion:**

Our findings demonstrated that YJJQE is estimated to effectively alleviate the depressed mood of patients with PSD by promoting the efficiency in information transmission of network functional connectivity and its integration ability in different brain regions. Therefore, the YJJQE would be useful as a non-pharmacological treatment to prevent PSD.

**Clinical trial registration:**

[http://www.chictr.org.cn/showproj.aspx?proj=55789], identifier [ChiCTR2000035588].

## Introduction

Globally, a stroke has been the second leading cause of death (Medeiros et al., 2020). From 1990 to 2019, the absolute number of incident strokes increased by 70.0%, prevalent strokes by 85.0%, and disability-adjusted life-years due to stroke by 3.20% (GBD 2019 Stroke Collaborators., 2021). Approximately one-third of stroke survivors developed poststroke depression (PSD), and the frequency is highest in the first year after a stroke (Virani et al., 2021). Previously, several investigators attempted to prevent the development of poststroke depression without success (Starkstein and Hayhow, 2019). Conversely, conventional antidepressants of pharmacotherapy may result in more side effects in increasing the risk of cardiovascular disease and stroke in PSD patients (Rasmussen et al., 2003; Almeida et al., 2006; Loubinoux et al., 2012; Swartz et al., 2016; MacQueen et al., 2017; Kapoor et al., 2019; Legg et al., 2019). Therefore, there is an urgent need for non-pharmacological treatments that are effective, safe, and easy to promote.

In recent years, there has been increasing clinical evidence of using traditional Chinese Tai Chi Qigong exercise to alleviate the symptoms of PSD (Lyu et al., 2021). Yijinjing Qigong exercise (YJJQE), which is similar to Tai Chi in China, but easier to learn, is a multicomponent traditional Chinese mind–body practice that combines meditation with slow, gentle, stretching muscle movements, deep diaphragmatic breathing, and relaxation (Guo et al., 2018; Chen et al., 2019; Yeung et al., 2019; Xing et al., 2020). Previous studies have shown that Yijinjing can effectively strengthen the muscles and ligaments around the spine, improve flexibility and balance, and help regulate a person’s physical and mental states (Xiang et al., 2006; Gao et al., 2021). We have proven that YJJQE can improve psychological well-being by reducing stress, anxiety, depression, and mood disturbance to manage KOA (Zhang D. et al., 2021; Zhang S. et al., 2021). However, no RCTs have been conducted specifically using YJJQE to evaluate its therapeutic benefits for those with PSD.

It is important to quantitatively analyze the neurological and psychiatric states of patients with PSD in order to develop personalized treatment plans that maximize outcomes. Quantitative electroencephalography (EEG) may also be a reliable instrument for detecting alterations of interhemispheric interaction poststroke that provide the functional electrical activity of neuronal assembles in the brain, including the integrity of the afferent sensory tract and the corticospinal tract as well as local and network electrical activity in the cortex (Kuriakose and Xiao, 2020; Vatinno et al., 2022). PSD has been associated with a dysregulation in the brain’s ability of cell communication (Leuchter et al., 2015). The functional brain network randomization linked to depressive disorders shows a low characteristic path length due to damage in the “core nodes” of the mood-related brain areas, leading to “disconnection” symptoms in the mood regulation pathway and ultimately reducing the operation efficiency of the whole brain network (Brakowski et al., 2017; Yu et al., 2019).

Overall, the objective of this study was to explore the efficacy of traditional Chinese Qigong exercise on PSD and quantitatively analyze the neurological and psychiatric mechanisms underlying EEG characteristics.

## Materials and methods

### Study design

This single-blinded, randomized controlled trial was approved by the Ethics Committee of Yue Yang Hospital of Integrated Traditional Chinese and Western Medicine Affiliated with Shanghai University of Traditional Chinese Medicine (project number: 2019-125), and registered in the Chinese Clinical Trial Registry (ChiCTR2000035588), and conducted in the same hospital in accordance with the Declaration of Helsinki. The study procedure and results were reported according to the CONSORT checklist. All participants gave us informed consent before inclusion.

### Participants

A total of sixty patients were enrolled between December 2019 and December 2021 at the inpatient Department of Rehabilitation Medicine in Yueyang Hospital. We obtained informed consent prior to the baseline assessments for eligibility. Patients were required to satisfy the following inclusion criteria: (1) the PSD patients were diagnosed following the international classification of the central nervous system (G00-G09) and the standard of Diagnostic and Statistical Manual of Mental Disorders, Fifth Edition (Chen Zhiluo, 1995; Xiande, 1996; The Psychiatry Branch of the Chinese Medical Association, 2001); (2) patients’ age was > 60 years and <75 years; (3) stroke from cerebral infarction diagnosed between the first and eighteenth month *via* computed tomography or magnetic resonance imaging; and (4) consequent evaluation HAMD-24 score ≤ 28 points. Alternately, the exclusion criteria were as follows: (1) severe encephalopathy, epilepsy, gastrointestinal bleeding, and heart failure and those in the drug withdrawal stage that lead to life-threatening conditions; (2) medical history of psychiatric or neurological disorder, or being diagnosed with moderate or severe depression; (3) psychopharmacological drug consumption; (4) had a history of substance abuse or alcoholism; and (5) were unable to independently complete the treatment, the scale assessment, and EEG examination, as required by the protocol.

### Power and sample size

We estimated the sample size based on a comparison between the intervention and control groups (Xu et al., 2021; Xue et al., 2021), which was characterized by the improvement in the HAMD-24 scores as the primary outcome. Previous studies demonstrated that the mean ± standard deviation (SD) of the HAMD-24 score in the intervention group was 28.69 ± 4.16 and that in the control group was 26.22 ± 3.98. Considering the statistical power to be 90% (α = 0.05), with a 10% attrition rate, we calculated the sample size by applying the formula. Based on the above parameters, the minimum sample size per group was 30, and the recruitment of 60 participants was needed.

### Randomization and masking

A total of sixty patients were randomly assigned to the intervention group for YJJQE combined with routine rehabilitation training and control group for only routine rehabilitation training group in a 1:1 ratio. The patients were requested not to change their ordinary physical activity and start any new intervention programs. In this context, independent scholars (third party) generated 60 unique seven-bit codes using random number functions in SAS 9.4 version software (SAS Institute Inc., Cary, NC, United States), then randomly split them into two groups, sequentially numbered them, and placed them in a sealed and opaque envelope to ensure confidentiality. Accordingly, the patients were divided into either the intervention or control group by picking a code from the envelope. The assessors, who measured all the patients and collected the outcome data, were unaware of any assigned intervention. All statistical analyses were performed while maintaining the data masking.

### Intervention program

Both groups of routine segmental rehabilitation training treatments were performed by the same therapist who was required to have more than 10 years of professional experience. A Yijinjing Qigong master with over 15 years of practice and instruction experience taught all classes.

### Control group

#### Routine segmental rehabilitation training

The control group was given single routine segmental rehabilitation training therapy, which was performed in a 60 min/session and once a day for 3 consecutive weeks. Briefly, for the arms, the patient lay on the back and the therapist treated the dysfunctional side with posthemiplegia manipulation; for the trunk, the patient lay on the back, with knees/hips bent and both hands tucked around the knees and the therapist gently shook the patient’ s body to the left and right; and for the legs, the therapist stretched the iliopsoas, quadriceps, hamstrings, and calf triceps (Jiang et al., 2022).

### Intervention group

#### Yijinjing Qigong exercise+routine segmental rehabilitation training

**FIGURE 1:**
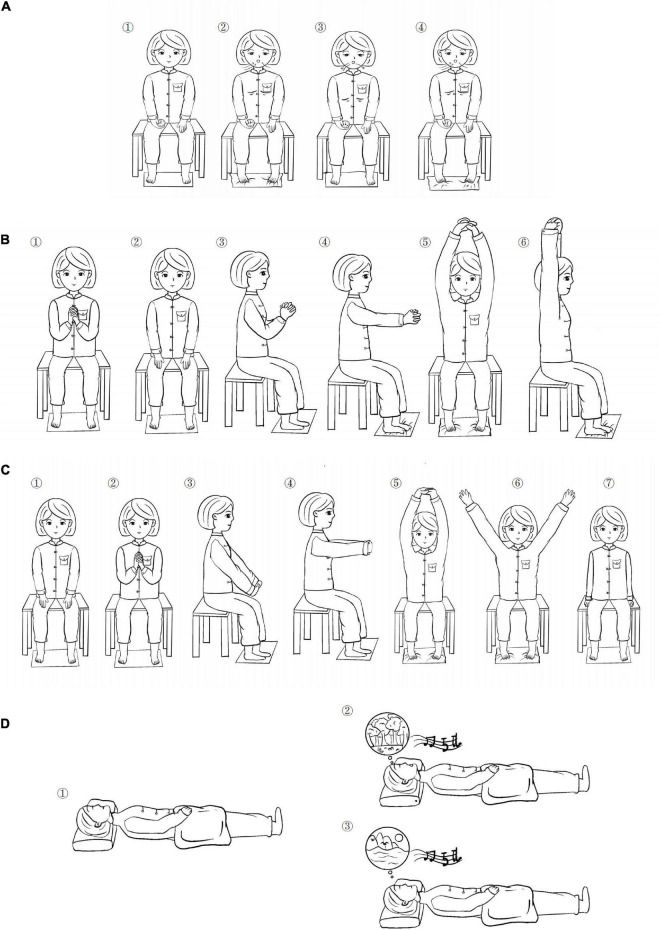
Qigong operation steps. **(A)** Action 1 Breathing practice; **(B)** Action 2 Sitting position Yijinjing (YJJ); **(C)** Action 3 Sitting position YJJ; and **(D)** Action 4 meditation and relaxation.

In addition to the routine segmental rehabilitation training, the patients were trained therapeutically with specifically tailored Yijinjing Qigong movements. The Qigong master displayed and guided the exercise action face to face for patients every day and the therapist helped these patients practice by driving the affected side with the healthy side. All the patients were taught in a group together. The patients in this group practiced a 60 min/session once a day for 3 consecutive weeks. Each session included a 10-min warm-up, 40-min practice, and refinement (explaining some theories and procedures of Qigong and training motion control for muscle and joint, and meditation and rhythmic breathing techniques), and a 10-min cool-down period (Figure 1 and Table 1).

**TABLE 1:** Details of the operation of Yijinjing Qigong exercise (YJJQE).

Sequence of details	Action details
Action 1	Let the patient warm up and adjust the mood and enter the exercise stage for 10 min, Subject is sitting in a stable chair or a wheelchair suitable for his position, the healthy hand held a timer, the affected hand remained relaxed, the abdominal wall is relaxed, focus on breathing deep and evenly; when inhaling, scratching toes, adjust the eyes in a relaxed state, attention to the chest and abdomen, position is kept for 10 s, and then exhale, relax the toes, feel deep breathing attention to the fluctuations of the chest and abdomen. While in the process, feel your scratching toes and press the timer, this phase of the operation also lasts for 10 s. Repeat for 15 min.
Action 2	Watching the video under the exercise training, subject sitting in a stable chair positions, steady center of gravity, tight abdomen, comfortable head, flat eyes, tongue against palate, dropped shoulder, elbow, knee, are kept at 50–90°, normal breathing, intertwined hands with 10 fingers crossed, the healthy hand drives the affected hand, continue to raise-up to the chest position, slowly stretch forward, arms raised, inhale for 10 s while scratching toes, relaxed shoulder, straight-up body, Continue to reach your hands toward your head and ears, keep balance for 10 s. Subject assessed according to the upper limb movement function with angle adjustment for 10 min.
Action 3	Repeat exercise in motion, attention to the change in the wrist, crossed fingers, down palms, keep stretch, steady center of gravity, tight abdomen, straight-up body, slowly forward arms and palms, inhale for 10 s, shoulder relaxed, hands continue to extend-up to the head, scratching toes, keep balance for 10 s. ease your arms down from the sides, repeat for 10 min. Subject assessed according to the upper limb movement function with angle adjustment.
Action 4	Subject in a supine position, Supported and inclusive environment, undisturbed place with a sense of security, quiet, then listen to a 15-mins musical meditation, body relaxed, knee joint in a comfortable state to relax, with soft blankets, warm, and comfortable lights, temperature, and volume, staff open the meditation music, to stabilize the emotions. breathing with closed eyes, with the sound prompt; in a soft music scene imagination (forest, flowers, and water), feel the fragrance of the sun, air, and grass, feel the breeze, the strength of the sea, close to nature and self-connection. Practice for 15 min.

### Outcome measurements

**FIGURE 2:**
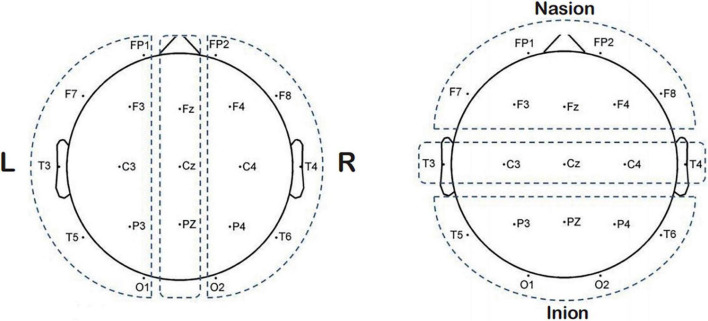
Electrode position diagram.

We assessed the therapeutic effects using the changes in the HAMD-24 score; a decrease in the score indicates an improvement in the depressive state. In addition, we used EEG to probe the effect of Qigong on brain activity. Digital EEG (EEG-9200K, Nihon Kogyo Co., Ltd. Huancheng North Road, Fengxian District, Shanghai) was used for EEG recording. The EEG data collection of patients is operated by a professional engineer according to the strict operation standard unified process. We placed the electrodes by the international 10–20 system standard and two reference electrodes were used, one on each earlobe. A 21-wire EEG was recorded; the corresponding of each recording electrode to its name was: FP1-FP2-F3-F4-C3-C4-P3-P4-O1-O2-F7-F8-T3-T4-T5-T6-Fz-Cz. The two reference electrodes were A1 (left ear) and A2 (right ear) (Figure 2). We kept all electrode impedances < 10 kΩ, and continuously recorded the signals at a sampling rate of 200 Hz. We amplified the EEG signal and divided the data into four frequency bands (delta: 1–4 Hz, theta: 4–8 Hz, alpha: 8–13 Hz, and beta: 13–30 Hz). Thereafter, the examination environment was quiet, at room temperature, with appropriate light, and the patient was not disturbed by vision and hearing. We asked the patients before the examination to not use psychotropic active substances and drugs (i.e., psychotropic drugs), nor another form of stimulant, and to have regular sleep the night before recording (Ríos-Herrera et al., 2019). About 2 h after the meal, we informed them about the purpose and scheme of the experiment. In the awake state during the whole test process, we asked the patients to avoid shaking their bodies. Noting that the electrodes should be far away from any interference source, such as power supply or electronic equipment, EEG data and signals were preprocessed using the EEG Matlab toolkit based on the Matlab software platform (R2012a, The Mathworks, Natwick, MA, United States). Spectrum analysis is a process used to quantify EEG. It decomposes a complex EEG signal into its component frequencies by applying the Fourier transform. The power spectrum reflects the amount of activity in the frequency band, and the relative power ratio can be used to assess the relative contribution of a specific frequency to the EEG signal (Cozac et al., 2016). The Phase Lag Index (PLI) was used to analyze the Phase characteristics of synchronous oscillation signals in different EEG bands in the exercise therapy group before and after treatment. After calculating the change of PLI between EEG lead pairs before and after treatment, the acute index was superimposed and averaged, and the global PLI was calculated. The PLI measures the asymmetry of the distribution of instantaneous phase differences between different EEG signals.


$$P\,L\,I=\left|\left\langle s\,i\,g\,n\,\left(\Delta \,\phi_{r\,e\,l}\,\left(t\right)\right)\right\rangle \right|=\left|\frac{1}{N}\,\sum_{n=1}^{N}s\,i\,g\,n\,\left(\Delta \,\phi_{r\,e\,l}\,\left(t_{n}\right)\right)\right|$$

where N represents the time point and φrel the phase difference of two-channel signals at time TN; the sign is a sign function, PLI values range from 0 to 1, where 0 indicates there may be no coupling, 1 indicates complete phase lock, and a higher value indicates stronger phase synchronization between the two signals (González et al., 2018).

Global efficiency (GE) reflects the information processing efficiency of the whole brain.


$$\frac{1}{N\,\left(N-1\right)}\,\sum_{i\neq j}\frac{1}{l_{i\,j}}$$

Local efficiency (LE) reflects the information processing efficiency of a local node (Kasakawa et al., 2016).


$$E_{l\,o\,c}\,\left(i\right)=\frac{1}{N_{G\,i}\,\left(N_{G\,i}-1\right)}\,\sum_{j,h\in G_{i}}\frac{1}{l_{j\,h}},$$

In this context, we documented the average LE and GE of each node in the brain network for the whole brain of every patient. Simultaneously, to analyze the brain network from the graph theory regarding PLI value as the strength, we generated the nodal topo-plots for each band *via* the digital EEG system (sparsity = 0.3) and the differences in the areas under the curve (AUC) under different sparsity degrees were determined.

### Data analysis

Categorical data were presented as percentages and compared by χ^2^ test; numerical variables were expressed as mean ± SD and compared between the two groups *via* a *t*-test. ANOVA of duplicate measurement data was used for observation of indicators at different time points, and multivariate analysis was used for comparison of the same observation index at different time points. The EEG data (GE or LE) were compared using the Wilcoxon rank-sum test. In a normal *t*-test or χ^2^ test, a *p*-value < 0.05 was regarded as statistically significant. For EEG analysis (four frequency bands), Matlab R2013b and its toolkit EEGLAB were applied, and we performed the Bonferroni multiple comparison corrections, wherein a *p*-value < 0.003 (P_*Bonferroni*_ = 0.05/16) was considered significant. Correlation analysis was performed first. If the scatter plot showed a linear distribution, and the data were normally distributed, the Pearson linear correlation test (Pearson correlation coefficient test) was performed. However, if the data did not show a normal distribution, correlation analysis using the Spearman correlation test was performed. If the scatter plot showed a non-linear distribution, a non-linear correlation analysis was performed.

## Results

### Participants and baseline characteristics

**FIGURE 3:**
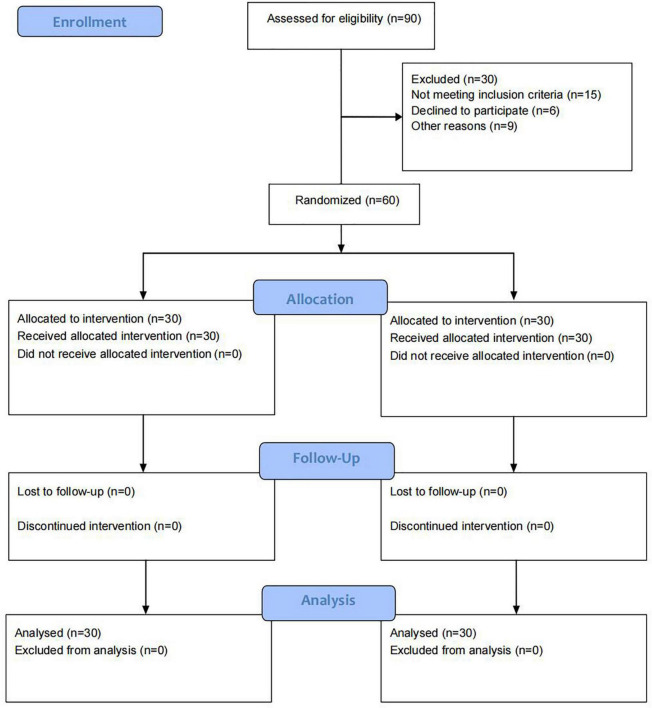
Recruiting process flowchart.

The flow diagram of the study is presented in Figure 3. A total of 90 patients were enrolled of which, 15 did not meet the inclusion criteria, six refused to participate in the study, and nine patients were unable to complete the entire study for various reasons. The remaining 60 patients met the eligibility criteria (30 in the Qigong group and 30 in the control group), and then completed the 3-weeks treatment and assessment, and 1 month of follow-up applying the HAMD-24 score. The EEG data of 49 patients before and after treatment were analyzed because the signal quality of seven in the control group and four in the treatment group was disturbed seriously by shaking of the small facial muscle group which interfered with the collection of signal in the EEG. No significant difference was observed between the two groups at the baseline as shown in Table 2 (*p* > 0.05). The average age of the patients in the control and Qigong groups was 65.37 ± 6.52 and 62.70 ± 8.63 years, respectively. In addition, there were no significant differences between the two groups in terms of body mass index, smoking, and alcohol drinking history, hypertension, diabetes, past cardiovascular disease, paraparetic side of the limb, disease duration, and stroke types (*p* > 0.05) (Table 2).

**TABLE 2:** General information of enrolled subjects.

Characteristics		Control (*n* = 30)	Qigong (*n* = 30)	χ^2^ or *t*	*p*
Sex (%)	Male	17 (56.67%)	17 (56.67%)	0	1
	Female	13 (43.33%)	13 (43.33%)		
Age		65.23 ± 6.29	62.03 ± 7.37	1.809	0.076
BMI		24.91 ± 3.23	24.82 ± 3.46	0.107	0.915
Smoking example (%)	Yes	8 (13.33)	9 (15.00)	0.082	0774
	No	22 (36.67)	21 (35.00)		
Alcohol drinking example (%)	Yes	12 (20.00)	14 (23.33)	0.271	0.602
	No	18 (30)	16 (26.67)		
Hypertension		4/26	2/28	0.741	0.671
Diabetes		12/18	15/25	0.606	0.436
Past cardiovascular disease		28/2	18/12	1.2	0.273
Paraparetic side of the limb	Left	18	15	0.606	0.436
	Right	12	15		
Disease duration (m)		7.53 ± 4.09	7.97 ± 5.27	0.356	0.723
Stroke type	Infarct	26 (86.67)	23 (76.67)	1.002	0.317
	Hemorrhagic	4 (13.33)	7 (23.33)		

### Effect of Qigong on HAMD-24 score

The effect of Qigong on the HAMD-24 score was assessed at the baseline after 3-weeks and 1-month follow-up treatment. Table 3 showed that the HAMD-24 scores at the baseline were similar. The Qigong groups can effectively improve depression at different times. Over time, the effect became much more obvious. The patients were followed up for 1 month, whereas the average score significantly decreased in the Qigong group compared with the control group (9.87 ± 4.27 vs. 16.37 ± 4.95, *p* < 0.001). This outcome proved that YJJQE might have positive clinical effectiveness in improving PSD.

**TABLE 3:** HAMD-24 scores at the baseline 3 weeks and follow-up 1 month after treatment.

Scale		Control (*n* = 30)	Qigong (*n* = 30)	*f*	*p*
HAMD-24	Baseline	16.23 ± 4.19	14.57 ± 4.66	2.121	0.151
	3 weeks	16.60 ± 4.55	12.10 ± 4.75*^#^	14.028	<0.001
	1 month follow-up	16.37 ± 4.95	9.87 ± 4.27*^#^	29.633	<0.001

^#^Tip: Statistically significant using the repeated measurements comparison.

*Statistically significant using the multivariate analysis.

### Effect of Qigong on the electroencephalography signal features

As shown in Table 4, we compared the AUC for GE and LE regarding each sparsity using the four bands and evaluated the overall difference in AUC between the two groups. θ wave was significantly reduced after treatment compared with the control group (0.164 ± 0.005 vs. 0.158 ± 0.006; *p* < 0.001) and (0.099 ± 0.018 vs. 0.121 ± 0.013; *p* < 0.001). GE and LE brain network efficiency increased compared to the control group; the YJJQE group showed a significantly greater functional connectivity in the δ, θ, and β frequency bands in the brain network of the degree of phase synchronization (*p* < 0.001).

**TABLE 4:** Areas under the curve (AUC) of global efficiency (GE) and local efficiency (LE) associated with different sparsity levels in the electroencephalography (EEG) examination.

EEG feature		Control (*n* = 23)	Qigong (*n* = 26)	*t*	*p*
GE-δ	Baseline	0.164 ± 0.005	0.165 ± 0.003	1.061	0.294
	3 weeks	0.162 ± 0.007	0.157 ± 0.010	2.061	0.045*
GE-θ	Baseline	0.160 ± 0.007	0.156 ± 0.008	2.061	0.045
	3 weeks	0.158 ± 0.006	0.164 ± 0.005	3.771	<0.001*
GE-β	Baseline	0.164 ± 0.006	0.165 ± 0.005	0.419	0.677
	3 weeks	0.161 ± 0.006	0.158 ± 0.007	1.868	0.068*
GE-α	Baseline	0.146 ± 0.009	0.139 ± 0.011	2.346	0.023
	3 weeks	0.151 ± 0.010	0.144 ± 0.014	2.051	0.046
LE-δ	Baseline	0.116 ± 0.015	0.112 ± 0.002	0.769	0.446
	3 weeks	0.119 ± 0.018	0.131 ± 0.022	2.033	0.046*
LE-θ	Baseline	0.115 ± 0.018	0.121 ± 0.018	1.185	0.242
	3 weeks	0.121 ± 0.013	0.099 ± 0.018	4.993	<0.001*
LE-β	Baseline	0.117 ± 0.016	0.139 ± 0.011	2.332	0.024
	3 weeks	0.120 ± 0.020	0.122 ± 0.175	0.284	0.778
LE-α	Baseline	0.115 ± 0.016	0.132 ± 0.016	3.175	0.004
	3 weeks	0.128 ± 0.017	0.144 ± 0.020	2.912	0.006**

*Statistically significant using the Bonferroni multiple comparison. **p < 0.01 significant statistical differences were observed.

**FIGURE 4:**
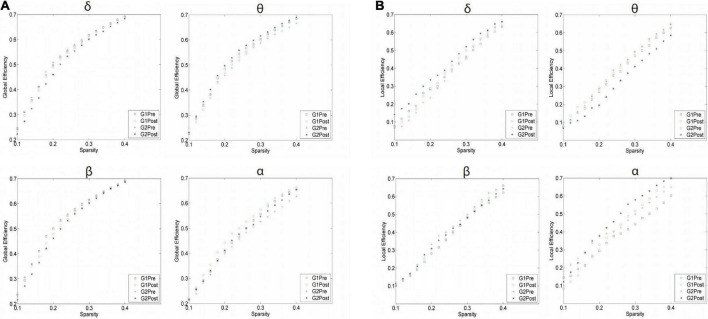
Difference of topological parameters of global efficiency between Qigong group and control group in **(A)** Delta band; Theta band; Beta band; and Alpha band before and after treatment. The sparsity was set as 0–80%. Delta band: δ; Theta band: θ; Beta band: β; and Alpha band: α. Difference of topological parameters of local efficiency (LE) between Qigong group and control group in **(B)** Delta band δ, Theta band θ, Beta band β, and Alpha band α before and after treatment. The sparsity was set as 0–80%.

**FIGURE 5:**
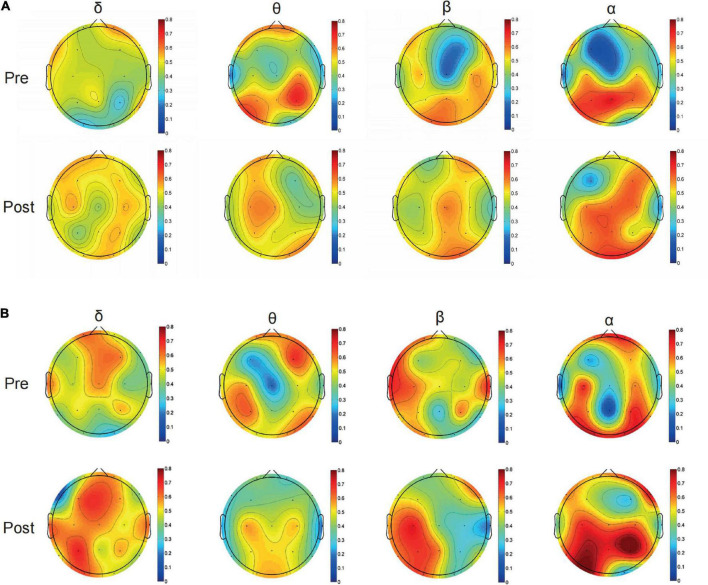
Typical topographical maps of power density [using the average local efficiency (LE)] Delta band, Theta band, Beta band, and Alpha band of control group **(A)** and Qigong group **(B)** after treatment, the frequency spectrum of (Fp1.Fp2.F3.F4), (T3.T4.T5), (P3) are mainly different in δ band; the frequency spectrum of the whole brain is different in θ band; and (T3.T5), (C3), (P3.Pz), (O1), and a small part (F8) are different in β band; the frequency spectrum of (FP1.FP2), (P3), and (O1.O2) are different in α band.

**FIGURE 6:**
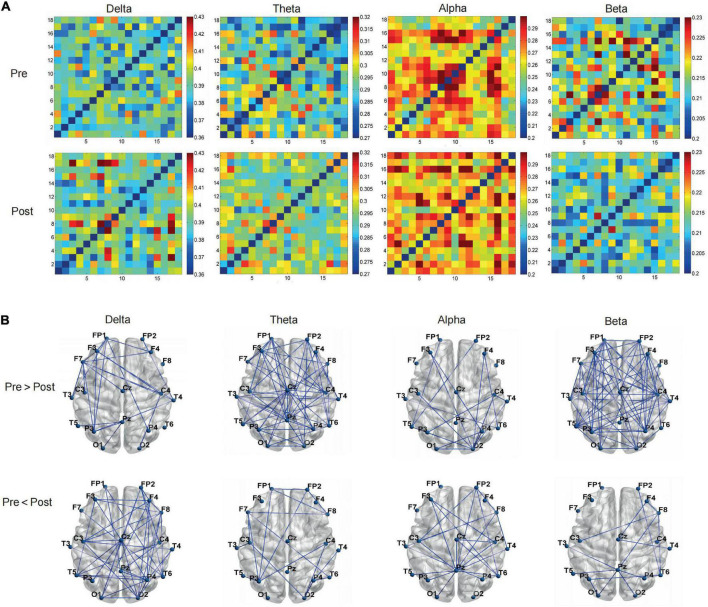
The Qigong group **(A)** by regarding phase lag index (PLI) value as the strength, verified 18*18 PLI functional connection moments, calculated global, and local topological parameters **(B)** on the brain network from the graph theory.

The curves of GE and LE regarding different sparsity levels are presented in Figure 4, and for the 2o groups each wave band, the nodal topo-plots constructed using average LE are shown in Figure 5. Before treatment, the θ wave mostly appeared in the right frontal lobe, frontal lobe, left frontal lobe, right temporal lobe, right anterior temporal lobe, left posterior temporal lobe, and left parietal lobe. The PSD patients reduced the power of the right temporal lobe in the prefrontal cortex (FP2.FP1.F4), which reduced the length of the feature path, accelerated the information exchange between neighbor nodes, improved the speed of information transmission between brain networks, and improved the network efficiency. We continued to evaluate functional brain connections in different frequency bands before and after the Qigong group. We verified 18 × 18 PLI functional connection moments and calculated global and local topological parameters (Figure 6).

The average PLI of the brain functional connectivity in different frequency bands before and after the Qigong group practice was evaluated as follows: the δ frequency bands (0.3680 ± 0.0028 vs. 0.3718 ± 0.0066; *p* < 0.01), θ frequency bands (0.2755 ± 0.0039 vs. 0.2791 ± 0.0032; *p* < 0.001), and β frequency bands (0.2037 ± 0.0030 vs. 0.2006 ± 0.0020; *p* < 0.001). The sparsity was set as 30%, Statistics were performed for the electrical signals at each electrode and the brain network topology map was depicted. The results showed that the local efficiency of the δ frequency network was enhanced in the F7 left anterior temporal, O1 left occipital, left parietal P3 (0.4659 ± 0.0843 vs. 0.5204 ± 0.0854; *p* < 0.05). Global and local efficiency enhancement of θ band network in FP1, right prefrontal FP2, F3, F4, right frontal, central region of CZ, and right posterior temporal T6 was (0.5872 ± 0.0347 vs. 0.6159 ± 0.0231; *p* < 0.001), (0.4932 ± 0.0966 vs. 0.4112 ± 0.0824; *p* < 0.01). The local efficiency of the α frequency band network was significantly enhanced in F8 right anterior temporal, P3 left parietal lobe, PZ top, and midline, and O2 right occipital lobe (0.4989 ± 0.0694 vs. 0.5780 ± 0.0878 *p* < 0.001). The global efficiency enhancement of the β frequency band network was located in F7 left anterior temporal; PZ midline; and F8 right anterior temporal, T4 right middle temporal, and T6 right posterior temporal, (0.6161 ± 0.0184 vs. 0.6021 ± 0.0225; *p* < 0.01). Power density Comparison between the two groups showed statistical significance in the enhancement of alpha frequency power density (*p* < 0.05). The changes in δ, θ, α, and β band power density in the two groups before and after treatment are shown in Figures 9, 10.

**FIGURE 7:**
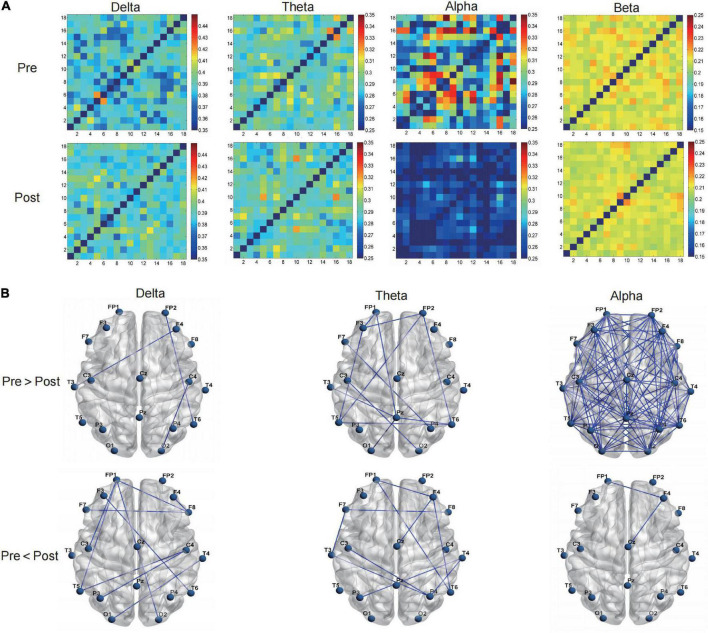
The control group **(A)** by regarding phase lag index (PLI) value as the strength, verified 18*18 PLI functional connection moments, calculated global, and local topological parameters **(B)** on the brain network from the graph theory.

**FIGURE 8:**
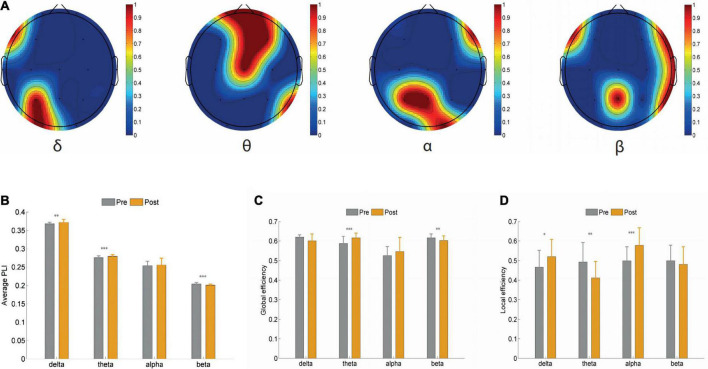
**(A)** Global efficiency (GE) local efficiency (LE) of each frequency band was evaluated when the average phase lag index (PLI) sparsity of brain functional connections **(B)** different frequency bands was equal to 0.3 before and after Qigong group, Delta band, Theta band, Alpha band, and Beta band. **(C)** The sparsity was set as 30%, GE of each frequency band was evaluated. **(D)** The sparsity was set as 30%, LE of each frequency band was evaluated. *P < 0.05, have statistical differences. **P < 0.01, significant statistical differences. ***P < 0.001, significant statistical differences.

**FIGURE 9:**
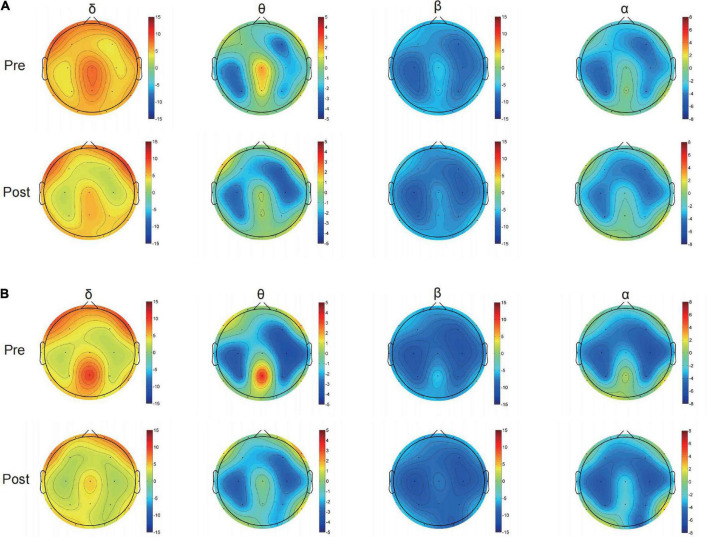
Power spectral density before and after treatment in the **(A)** control and **(B)** Qigong groups.

**FIGURE 10:**
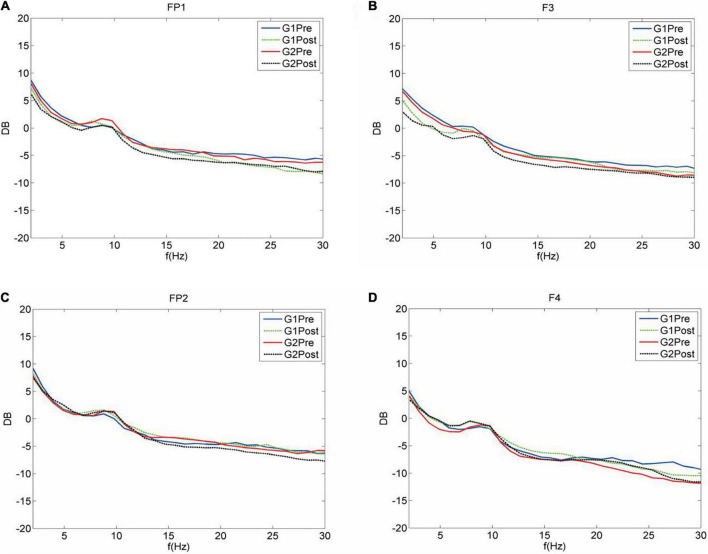
Average spectrum curves of the full leads. The average spectrum curves of both groups were significant before and after treatment (Fp1.Fp2.F3.F4). Delta band (1–4 Hz); theta band (4–8 Hz); alpha band (8–13 Hz); and beta band (13–30 Hz). **(A)** FP1 G1 of the control group before treatment. The values for the delta and theta band power increased, whereas, those for the alpha band power decreased. However, the alpha band power increased after treatment. G2 before treatment of the treatment group. Increasing trend in the powers of four frequency bands. The powers of the four frequency bands decreased after treatment. **(B)** F3 left frontal lobe G1 of the control group before treatment, Delta, theta, alpha, and beta bands showing an increasing trend in power. After treatment, the delta and beta bands showed a decay of power, whereas the theta band power increased. G2 of the treatment group. The powers of four frequency bands showing an increasing trend. The powers of the four frequency bands decreased after the treatment. **(C)** FP2 G1 of the control group before the treatment. The alpha band power showed an increasing trend, whereas the powers of the delta, theta, and beta bands showed a decreasing trend. After treatment, the alpha and beta band powers increased. G2 of the Qigong group showed a delta power coast down and increases in the delta, alpha, and beta powers. After treatment, the delta power increased, whereas the theta and beta powers showed a coast down. **(D)** F4 G1 of the control group before the treatment showed decreases in the delta, theta, alpha, and beta band powers. After treatment, the delta and beta bands showed a decay of power, whereas the alpha band power increased. G2 of the Qigong group showed decreases in the delta, theta, and beta band powers. After treatment, the delta, theta, and beta band powers increased.

## Discussion

To the best of our knowledge, this is the first randomized, controlled study to demonstrate that YJJQE can alleviate the depressed mood of patients with PSD after 3 weeks of intervention. The findings showed that the YJJQE after treatment resulted in significant improvements in mental and physical anxiety symptoms, such as restlessness, sweating, dry mouth, sleep quality (falling asleep became easier and the number of night awakenings was also reduced), and physical symptoms of fatigue and gastrointestinal disorders. Our study presented the YJJQE as a mind–body intervention to be useful as a non-pharmacological treatment to prevent PSD.

Previous studies on the rehabilitation of patients after stroke patients through traditional Chinese exercise have shown that they can enhance the ability of patients to control their trunk as well as leg strength and mobility. Traditional Chinese exercise can also improve the function of respiratory muscles and significantly improve patients’ cognition and quality of life (Zhang et al., 2016; Yuen et al., 2021). Meanwhile, in another study on poststroke mind–body problems, acupuncture combined with YYJQE revealed superior improvement effects on the motor function scores and self-rated depression scale scores. To date, previous studies of evidence-based Qigong exercises for treating PSD were extremely limited (Thomas et al., 2019; Hordacre et al., 2021). One meta-analysis has been proposed but with a lack of evidence concerning its effects conclusion (Dong et al., 2020). Nevertheless, Qigong is one of the effective complementary therapies for treating depression (Haller et al., 2019). Qigong could improve the self-rating depression scale and anxiety scale scores, which exert auxiliary effects on improving lung function in patients with COPD (Wu et al., 2019). In addition, several studies have suggested its potentially beneficial effect on symptoms of anxiety among individuals with drug abuse (Liu et al., 2020). However, several studies demonstrated no benefits of Qigong in treating depression; thus, further research using randomized controlled trials and rigorous designs is needed (Oh et al., 2013.)

This study indicated that the YJJQE treatment showed a significant reduction in θ wave and an increase in the GE and LE of the network functional connectivity in different brain regions by EEG. A recent study evaluating the brain network performance of patients with PSD using EEG mutual information showed significantly weakened connections between the left and right cerebral hemispheres, and this feature was more obvious with an increasing degree of depression (Sun et al., 2018; Xu et al., 2019). The right PFC is more related to negative emotions. An injury of the right PFC leads to a lack of the ability to experience positive emotions, even depressive symptoms (Morris, 1997). We found that PSD patients reduced the strength of the right temporal lobe in the prefrontal cortex [(FP2), FP1.F4], enhanced connectivity in the left prefrontal cortex, and improved the network efficiency after treatment. At a sparsity level of 0.3, the θ waves in our study showed the most significant changes in the prefrontal cortex and the right temporal lobe and significantly enhanced functional brain connectivity in the Qigong group. These regions are associated with the regulation of emotion, motivation, learning, attention, social behavior, and behavioral decision-making. The brain activity shows that the interconnection between the nodes changed due to the Qigong meditation training. It was attributed to relaxation, concentration, and behavior control. Moreover, θ waves are also involved in the edge-PFC interaction and the synchronization of brain network activities. The interaction between the above regions can be mediated by the θ waves, which support the important communication mechanisms between memory, executive function, and depression-related brain regions.

At a sparsity level of 0.3, the β wave decreased, the α and δ waves increased, and the network efficiency was significantly increased in the Qigong group. Previous studies found that within 3–6 months after the stroke, the EEG abnormalities in PSD patients showed a decreased α frequency and a slow wave increase in the frontal lobe (Coito et al., 2019), which have been considered independent predictors of PSD (Zheng et al., 2018). The α and δ low-frequency activity of the wave, as well as the anomalous oscillations, have an asymmetry between the left and right hemispheres (Pierre et al., 2004; Wang et al., 2017; Rochais et al., 2018), β wave increasing in bilateral or right frontal waves in patients with depression, the anxiety-type depression group in elderly depressed patients showed an enhanced β 1-wave activity in the parietal and occipital regions (Suzuki et al., 1996; Volf and Passynkova, 2002; Pizzagalli et al., 2018). The association between low β2 main peak frequency in the EEG and the vulnerability to depression has also been noticed in animal models (Claverie et al., 2016).

**FIGURE 11:**
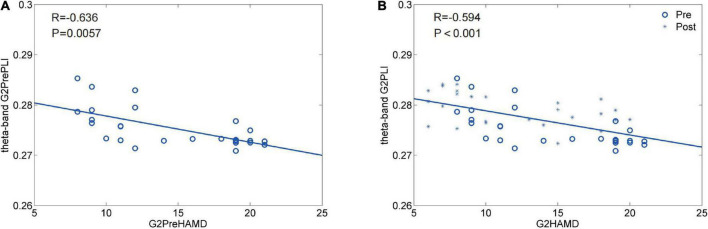
**(A,B)** HAMD-24 score and EEG correlation analysis before and after treatment in both groups. G2 of the Qigong group Theta band pretherapy, By After correction *p* = 0.0057; It has relevance, *R* = −0.636104330651571 negative correlation. G2 of the Qigong group Theta band post-treatment, By After correction *p* = −8.446060534953326e-05, There was a significant correlation. *R* = −0.593536751161075 had a negative correlation.

In this study, the HAMD-24 scores and EEG correlation analysis in the Qigong group, which had a negative correlation, indicated that depression decreases with increase in Qigong training; those in the control group were not correlated (Figure 11). The control group was not correlated. The EEG data strongly supported the results of the HAMD-24 score, which indicated that Qigong could enhance the GE-θ of the brain network in patients with PSD while homogenizing the LE-θ. Although there has been published evidence, the link between the θ band rhythm and PSD was not fully revealed. Our result is logically consistent with the recognized knowledge that enhanced θ-activity and symmetry are associated with better outcomes of PSD. The improvement of the θ frequency band preliminarily indicates that practicing the modified YJJQE (focusing on breathing and limb movements) during meditation could enhance brain network activity, improve the efficiency of information communication/integration between different brain areas, and alleviate negative emotions. This exercise could be widely applied and further studied in relation to the mechanism of brain connectivity.

This study has several limitations. Due to the small sample size, we compiled patients with different locations of stroke lesions to analyze EEG, yet it is ambiguous whether this might impact the results of the brain network topology; further studies are required to screen patients with a lesion in a specific area to confirm our conclusion. The period of intervention was only 3 weeks, and it remains unclear whether long-term intervention could also provide better efficacy.

## Conclusion

This is the first randomized controlled study to use a YJJQE training protocol specifically designed for patients with PSD and determine the mechanism of network functional connectivity in different brain regions by EEG. The findings added increasing evidence regarding the clinical benefits of Yijinjing Qigong as a non-pharmacological therapeutic modality for patients with PSD to improve depressive symptoms. The PSD patients after YJJQE intervention reduced the length of characteristic path, and accelerated the information exchange between neighbor nodes, and improved the transmission speed of information and the network efficiency between brain networks. Therefore, this study provides a non-drug therapy for the treatment of PSD. Further evidence is needed to support the conclusions of this exploratory study.

## Data availability statement

The raw data supporting the conclusions of this article will be made available by the authors, without undue reservation.

## Ethics statement

Written informed consent was obtained from the individual(s) for the publication of any potentially identifiable images or data included in this article.

## Author contributions

PS, SZ, LJ, CY, ZM, QZ, and MF drafted, reviewed, and revised the manuscript. QZ and MF managed the methodology of the study. All authors have read and approved the theory and manuscript of the article.
